# Prevalence of posterior staphyloma and factors associated with its shape in the Japanese population

**DOI:** 10.1038/s41598-018-22759-y

**Published:** 2018-03-15

**Authors:** Shogo Numa, Kenji Yamashiro, Tomotaka Wakazono, Munemitsu Yoshikawa, Masahiro Miyake, Hideo Nakanishi, Akio Oishi, The Nagahama Study Group, Takahisa Kawaguchi, Kazuya Setoh, Yoshimitsu Takahashi, Shinji Kosugi, Takeo Nakayama, Yasuharu Tabara, Fumihiko Matsuda, Nagahisa Yoshimura, Akitaka Tsujikawa

**Affiliations:** 1 Department of Ophthalmology and Visual Sciences, Kyoto University Graduate School of Medicine, Kyoto, Japan; 2 Department of Ophthalmology, Otsu Red Cross Hospital, Shiga, Japan; 3 Center for Genomic Medicine/Inserm U.852, Kyoto University Graduate School of Medicine, Kyoto, Japan; 4 Department of Health Informatics, Kyoto University Graduate School of Medicine, Kyoto, Japan; 5 Department of Medical Ethics and Medical Genetics, Kyoto University Graduate School of Medicine, Kyoto, Japan

## Abstract

Myopia is increasing rapidly worldwide. We performed a cross-sectional study to investigate the prevalence of posterior staphyloma, a complication of myopia, and its shape characteristics in relation to age, sex, and axial length (AL) in a Japanese community-based cohort. The right eyes of 3748 participants who underwent fundus photography and optical coherence tomography (OCT) examination were evaluated. Posterior staphyloma prevalence was evaluated using fundus photographs and OCT images. Furthermore, fundus shapes were analyzed by measuring local fundus curvatures on 6 mm cross-line OCT images at intervals of 1 µm. The mean and variance of the curvatures were calculated to represent the fundus shape of each eye for investigation of the relationship between fundus curvature and age, sex, and AL. Seventy-seven eyes (2.05%) had posterior staphyloma. The mean and variance of the fundus curvatures were significantly greater in women than in men and became greater with age, suggesting that the shape of the staphyloma was steeper and less smooth in women and elderly subjects. AL and mean curvature showed a significant correlation (*P* = 2 × 10^−16^, *R* = 0.480), which was significantly affected by age (*P* < 2 × 10^−16^). Quantitative analysis of fundus shapes was useful for statistical analysis of posterior staphyloma in relation to age, sex, and AL.

## Introduction

The prevalence of myopia and high myopia has increased markedly worldwide, especially in East Asia^1–6^. Several large cohort studies in Asia, including the Tajimi Study in Japan^7^, the Beijing Eye Study in China^8^, and the Shihpai Eye Study in Taiwan^2^, showed that myopic macular degeneration in high myopia was a leading cause of legal blindness. In Western countries, myopic macular degeneration has been reported to be the second to fourth most frequent cause of blindness^9–11^. Formation of posterior staphyloma in high myopia is a key component of a spectrum of vision-threatening myopic maculopathies^12–14^. Many studies have shown that retinoschisis, retinal detachment, macular hole, choroidal neovascularization, and chorioretinal atrophy occur in association with posterior staphyloma^15–18^. Recently, quantitative analysis of optical coherence tomography (OCT) images revealed detailed correlations between the shape of posterior pole fundus/staphyloma and the development of complications in highly myopic eyes^19,20^. Even though knowledge of posterior staphyloma is essential for understanding the complications of high myopia, its prevalence has not been thoroughly investigated.

Population-based cohort studies of the prevalence of posterior staphyloma are limited. The prevalence was reported to be 0.7% in the Blue Mountains Eye Study, 0.8% in the Handan Eye Study, and 1.6% in the Beijing Eye Study^21–23^. Further, it was reported to be 0.1% among Singaporean children aged 12–16 years^24^ and 10.7% in patients with cataract in Saudi Arabia^25^. These reports suggest that the prevalence should be examined in association with age, sex, axial length (AL), and refractive error. Although the prevalence of posterior staphyloma in highly myopic eyes has been rarely examined in cohort studies, it could be calculated using data from previous reports on high myopia. The calculated prevalence of posterior staphyloma in highly myopic eyes varies from 19% to 90%, although most studies did not aim to evaluate its prevalence per se **(**Table S1**)**.

Posterior staphyloma is defined as “an outpouching of the wall of the eye that has a radius of curvature that is less than the surrounding curvature of the wall of the eye”^26^. This alteration of the curvature can be easily detected as the edge of staphyloma in the fundus images of ultra-wide field photography using devices such as the Optos scanning laser ophthalmoscope (Optos PLC, Dunfermline, Scotland, UK) or in the entire globe images of three-dimensional magnetic resonance imaging. However, in large cohort studies, such modalities are difficult to introduce in practice, and previous population-based studies utilized subjective clinical examination with ophthalmoscopy or fundus photographs to detect staphyloma.

Using OCT is another option to detect the alteration of posterior fundus curvature in eyes with staphyloma. Recent advances in OCT have improved detection of the detailed curvature of the fundus, and quantitative analyses of macula area OCT images yielded fairly correct diagnostic rates for staphyloma^19^. OCT would be of considerable help when investigating the prevalence of staphyloma and for quantitative evaluation of its posterior fundus shape in large cohorts. In the present report, we utilized both fundus photographs and OCT images to determine the prevalence of posterior staphyloma in the Japanese community-based cohort from the Nagahama Study and performed quantitative analysis of the OCT images to identify any correlations between posterior fundus shape of staphyloma and age, sex, and AL.

## Methods

The Kyoto University Graduate School and Faculty of Medicine Ethics Committee and the Nagahama Municipal Review Board of Personal Information Protection approved the study protocol and procedures used to obtain informed consent. All study procedures adhered to the tenets of the Declaration of Helsinki. All participants were fully informed about the purpose and procedures of the study, and written consent was obtained from each subject. Patient records and information were anonymized prior to analysis.

### Study participants

We examined the right eyes of 3866 participants at the 5-year follow-up of the Nagahama Study (Nagahama Prospective Genome Cohort for Comprehensive Human Bioscience). The baseline study examined 10,082 healthy Japanese individuals aged 30–74 years who were residing in Nagahama City between November 2008 and November 2010^27,28^. During the 5-year follow-up period, 8559 participants underwent examinations of AL (IOL Master, Carl Zeiss Meditec, Dublin, CA, USA) and spherical equivalent (ARK-530A, Nidek, Aichi, Japan), along with 45° color fundus photographs (CR-DG10, Canon, Tokyo, Japan) and a 6 mm cross line OCT scan (RS-3000, Nidek, Gamagori, Japan). Details of past cataract surgery, ocular surgeries other than cataract surgery, and ocular laser treatment, including photocoagulation, were obtained using a questionnaire. Of the 8559 participants examined between July 2013 and February 2016, the current study used 3866 participants examined between July 2013 and February 2015 who had no history of any ocular surgeries other than cataract surgery.

### Detection of posterior staphyloma

Staphyloma was diagnosed when localized ectasia of the sclera on the fundus photograph as in the previous cohort studies^21–23^ and an irregularly shaped Bruch’s membrane curvature at the ectasia on OCT images were observed. The detection was carried out by two retinal specialists (MM, HN). Any discrepancies were resolved by a third retinal specialist (KY).

### Analysis of fundus curvature

Fundus shapes were also analyzed quantitatively from 6 mm cross line OCT images using RetinaView software (Canon, Tokyo, Japan), which automatically plots the line of Bruch’s membrane on the OCT images and measures the local fundus curvature at intervals of 1 µm using every 3 sequential points 500 µm apart on the lines^19,20^. The software calculates the mean and variance of the absolute curvature using all measured values for local curvature. These two values were used to represent the shape of the fundus of each eye for further analysis.

### Detection of eyes with staphyloma by fundus curvature

To identify the best threshold values for the mean and variance of curvature and to be able to distinguish between eyes with or without staphyloma, receiver-operating characteristic (ROC) curves and areas under the curve (AUCs) were calculated by comparing the curvature values for eyes with staphyloma detected by fundus photographs and OCT images with those of eyes without staphyloma. To identify the best way of detecting eyes with staphyloma by using the curvature value(s), we evaluated four patterns as follows: mean curvature only, variance of curvature only, either mean curvature or variance of curvature, and both mean curvature and variance of curvature.

### Statistical analysis

The statistical analysis was performed using a freely available statistical software package (R for Mac OS X, version 3.2.3; The R Foundation for Statistical Computing, Vienna, Austria). The data are shown as the mean and standard deviation. The Fisher’s exact test and Mann-Whitney *U* test were used to compare proportions and continuous variables, respectively. Linear regression analysis was used to investigate the relationship between AL and mean absolute curvature. *P*-values < 0.05 were considered to be statistically significant.

## Results

Of the 3866 eyes enrolled, 3748 eyes of 3748 study participants (1193 men, 2555 women, mean age 57.3 ± 13.6 years) were analyzed. One hundred and eighteen eyes were excluded because the AL in the right eye was not measured or the sex and/or age of the study participant was not recorded (n = 97) or because the OCT images and/or color fundus photographs were of poor quality (n = 21). Only 3 of the 21 eyes with poor quality images were highly myopic (AL ≥ 26 mm). The mean AL of the 3748 eyes was 24.14 ± 1.42 mm, and 395 eyes (10.5%) were highly myopic (AL ≥ 26 mm; Table S2**)**. The men in the study were significantly older (59.4 ± 13.8 years vs 56.4 ± 13.4 years) and had a significantly longer AL (24.43 ± 1.35 mm vs 24.00 ± 1.43 mm) than the women (*P* = 6.03 × 10^−8^ and *P* = 2.71 × 10^−21^, respectively).

When evaluated using both fundus photographs and OCT images, 77 (2.05%) of the 3748 eyes examined had staphyloma **(**Table 1**)**. The prevalence of posterior staphyloma increased with age (0.53% in subjects aged 35–50 years, 1.5% in those aged 50–59 years, 2.6% in those aged 60–69 years, and 4.0% in those aged 70–79 years). The prevalence of posterior staphyloma was not significantly different between men and women in any age or AL group (*P* > 0.12). Posterior staphyloma was identified in 10.9% of the 395 highly myopic eyes. The prevalence of posterior staphyloma in highly myopic eyes also increased with advancing age (2.7% in subjects aged 35–50 years, 9.2% in those aged 50–59 years, 21.2% in those aged 60–69 years, and 43.6% in those aged 70–79 years).

**Table 1:** Prevalence of staphyloma in all study participants.

	Total	Men (n = 1193)	Women (n = 2555)	*P*-value (men vs women)
Staphyloma (+)	77/3748 (2.05%)	19/1193 (1.59%)	58/2555 (2.27%)	0.22
Age (years)				
≤49	7/1317 (0.53%)	2/367 (0.54%)	5/950 (0.53%)	1
50–59	8/529 (1.5%)	2/135 (1.5%)	6/394 (1.5%)	1
60–69	26/1006 (2.6%)	7/317 (2.2%)	19/689 (2.3%)	0.83
≥70	36/896 (4.02%)	10/374 (2.7%)	26/522 (5.0%)	0.12
Axial length (mm)				
<26	34/3353 (1.0%)	11/1034 (1.1%)	23/2319 (1.0%)	0.85
26–28	16/340 (4.71%)	3/137 (2.2%)	13/203 (6.4%)	0.12
28–30	25/53 (47.2%)	7/22 (31.8%)	18/31 (58.1%)	0.32
≥30	2/2 (100%)	0/0 (0%)	2/2 (100%)	—

**Figure 1:**
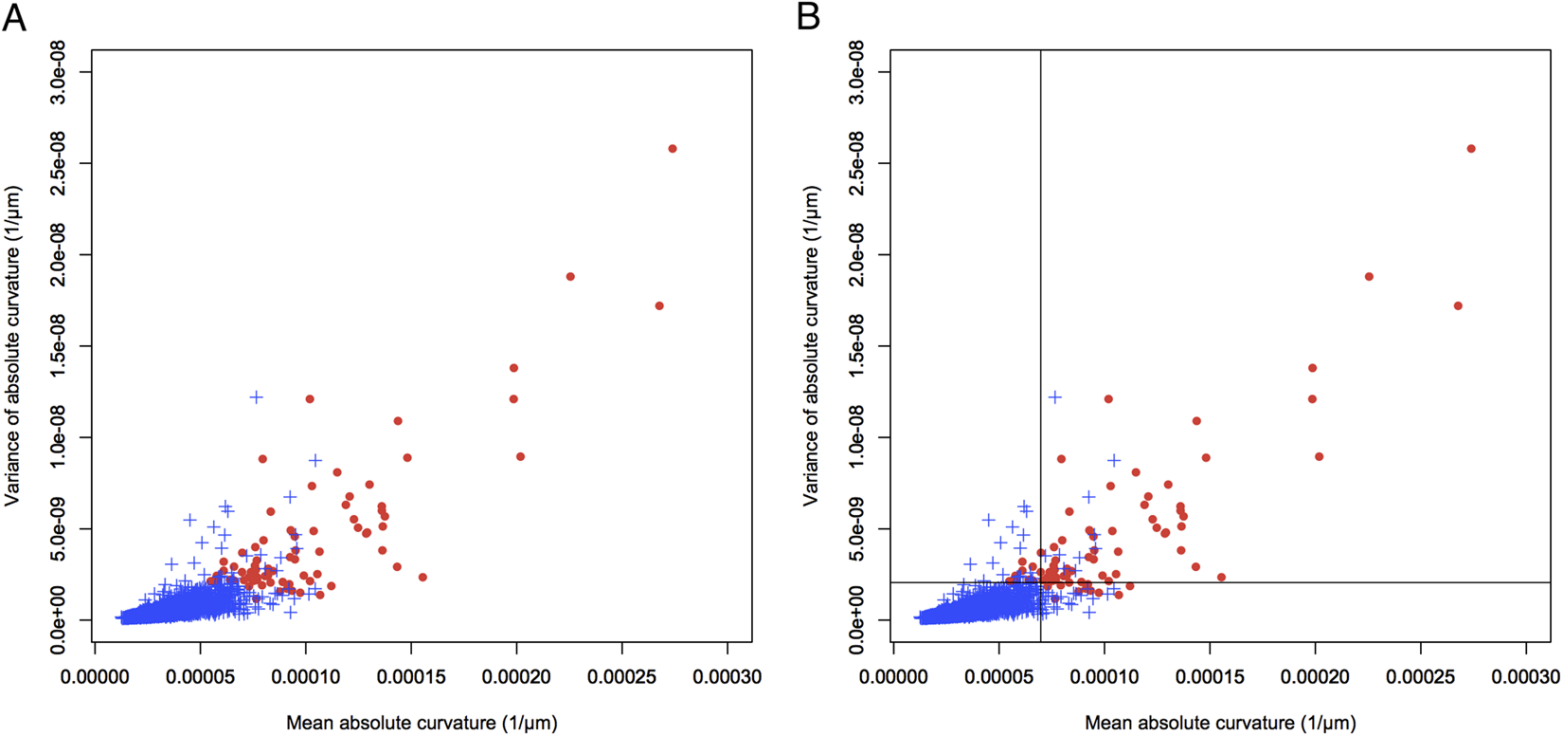
Scatter plot of all 3748 eyes assigning mean absolute curvature to the horizontal axis and variance of absolute curvature to the vertical axis. (**A**) Seventy-seven eyes with posterior staphyloma (filled red circle) and 3671 eyes without posterior staphyloma (blue cross). (**B**) Splitting lines at 6.97 × 10^−5^ in mean absolute curvature and 2.06 × 10^−9^ in variance of absolute curvature were added. Most of the eyes with staphyloma localize to the upper right segment of the splitting line.

Figure 1 plots the mean curvature and variance of curvature for the 3748 study eyes. Eyes with staphyloma had significantly greater values for mean curvature and variance of curvature than eyes without staphyloma (*P* < 2.96 × 10^-39^ and *P* = 5.13 × 10^−39^, respectively). Figure 1 also suggests that the cutoff value(s) for mean curvature and/or variance of curvature can divide eyes into those with and without staphyloma. Figure S1 shows the ROC curves for mean curvature and variance of curvature; the AUC was 0.994 for mean curvature and 0.994 for variance of curvature, suggesting that both parameters are well suited for detection of eyes with staphyloma. To obtain the highest correct detection rates for eyes with staphyloma, we sought to identify the cutoff value(s) for the following: mean curvature alone, variance of curvature alone, either mean curvature or variance of curvature, and both mean curvature and variance of curvature. The highest correct detection rates calculated were as follows: 3710/3748 (=99.0%) with a cutoff value of 7.24–7.26 × 10^−5^ or 7.34–7.39 × 10^−5^ in mean curvature; 3710/3748 (=99.0%) with a cutoff value of 2.03–2.06 × 10^−9^ in variance of curvature; 3711/3748 (=99.0%) with a cutoff value of 9.11–9.73 × 10^−5^ in mean curvature and a cutoff value of 2.06 × 10^−9^ in variance of curvature; and 3724/3748 (=99.4%) with a cutoff value of 6.97 × 10^−5^ in mean curvature and a cutoff value of 2.06 × 10^−9^ in variance of curvature. When we drew cutoff lines at 6.97 × 10^−5^ for mean curvature and 2.06 × 10^−9^ for variance of curvature, the correct detection rate for eyes with and without staphyloma was highest at 99.4%. With these cutoff lines, the sensitivity and specificity for detecting staphyloma were 84.4% and 99.7%, respectively.

**Figure 2:**
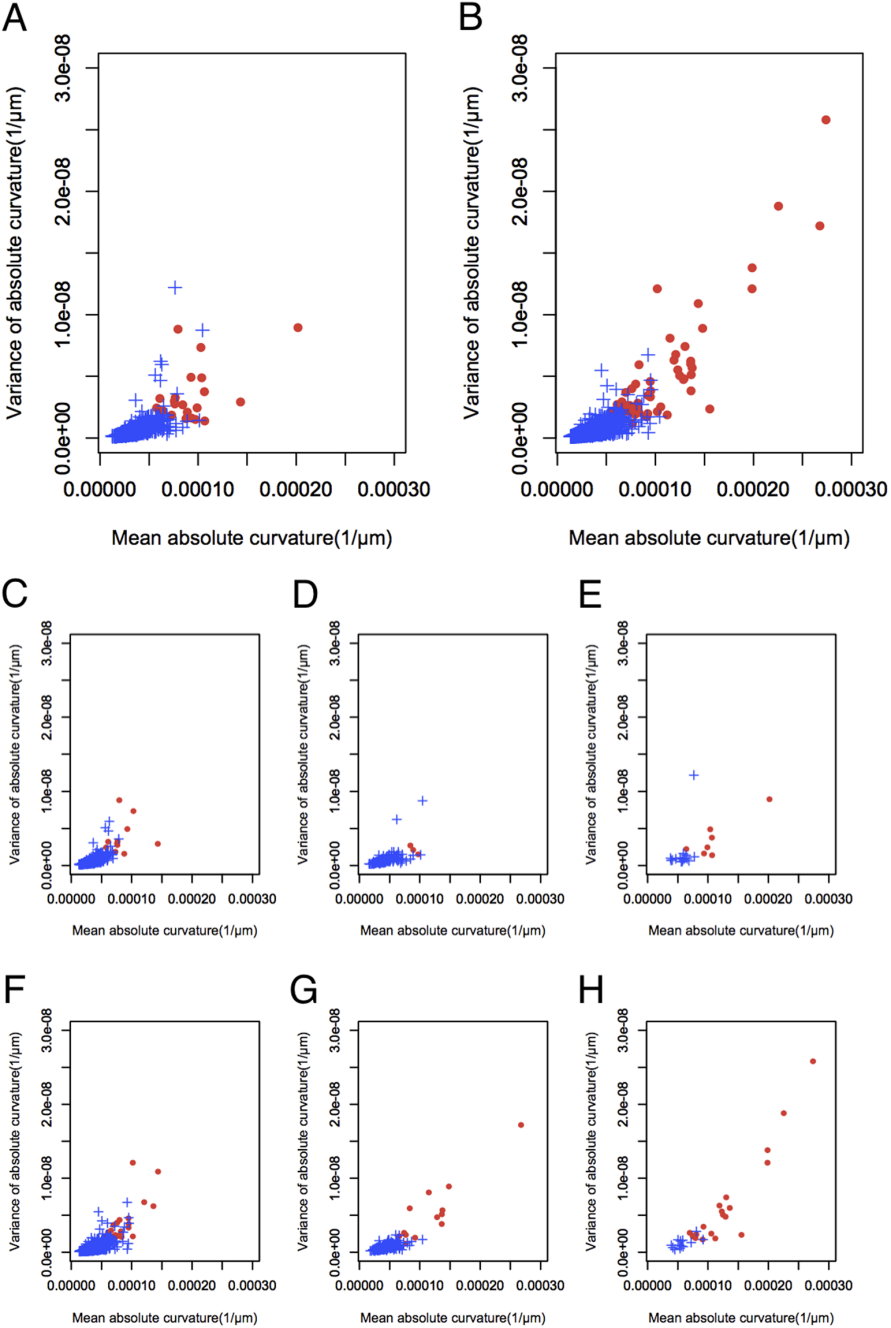
Scatter plot of (**A**) 1193 eyes from men and (**B**) 2555 eyes from women assigning mean absolute curvature to the horizontal axis and variance of absolute curvatures to the vertical axis. The scatter plots were stratified by axial length (AL) in eyes from men (C, AL < 26 mm; D, 26 mm ≤ AL < 28 mm; E, AL ≥ 28 mm) and eyes from women (F, AL < 26 mm; G, 26 mm ≤ AL ≤ 28 mm; H, AL ≥ 28 mm).

When the distribution of mean curvature and variance of curvature were plotted according to the sex of the subjects, it was clear that both values were higher in women than in men with staphyloma, and the posterior fundus curvature of eyes with staphyloma was more severe and had a less smooth surface in women than in men with high myopia regardless of the AL (Fig. 2).

**Figure 3:**
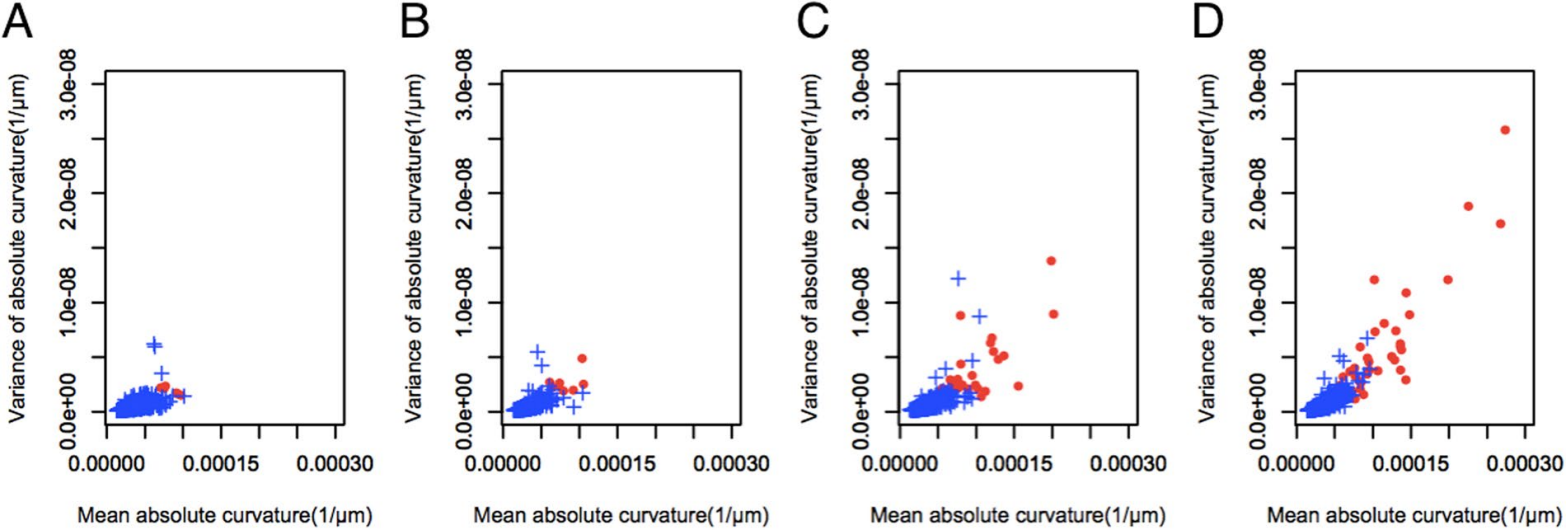
Scatter plot of (**A**) eyes in patients aged younger than 50 years, (**B**) eyes in patients aged 50–59 years, (**C**) eyes from patients aged 60–69 years, and (**D**) eyes in patients aged 70 years or older assigning mean absolute curvature to the horizontal axis and variance of absolute curvatures to the vertical axis.

When the distribution of mean curvature and variance of curvature were plotted according to the age of the subject, the values for both parameters increased with advancing age in eyes with staphyloma, indicating that the shape of the fundus becomes steeper and the surface less smooth with age in eyes with staphyloma (Fig. 3).

**Figure 4:**
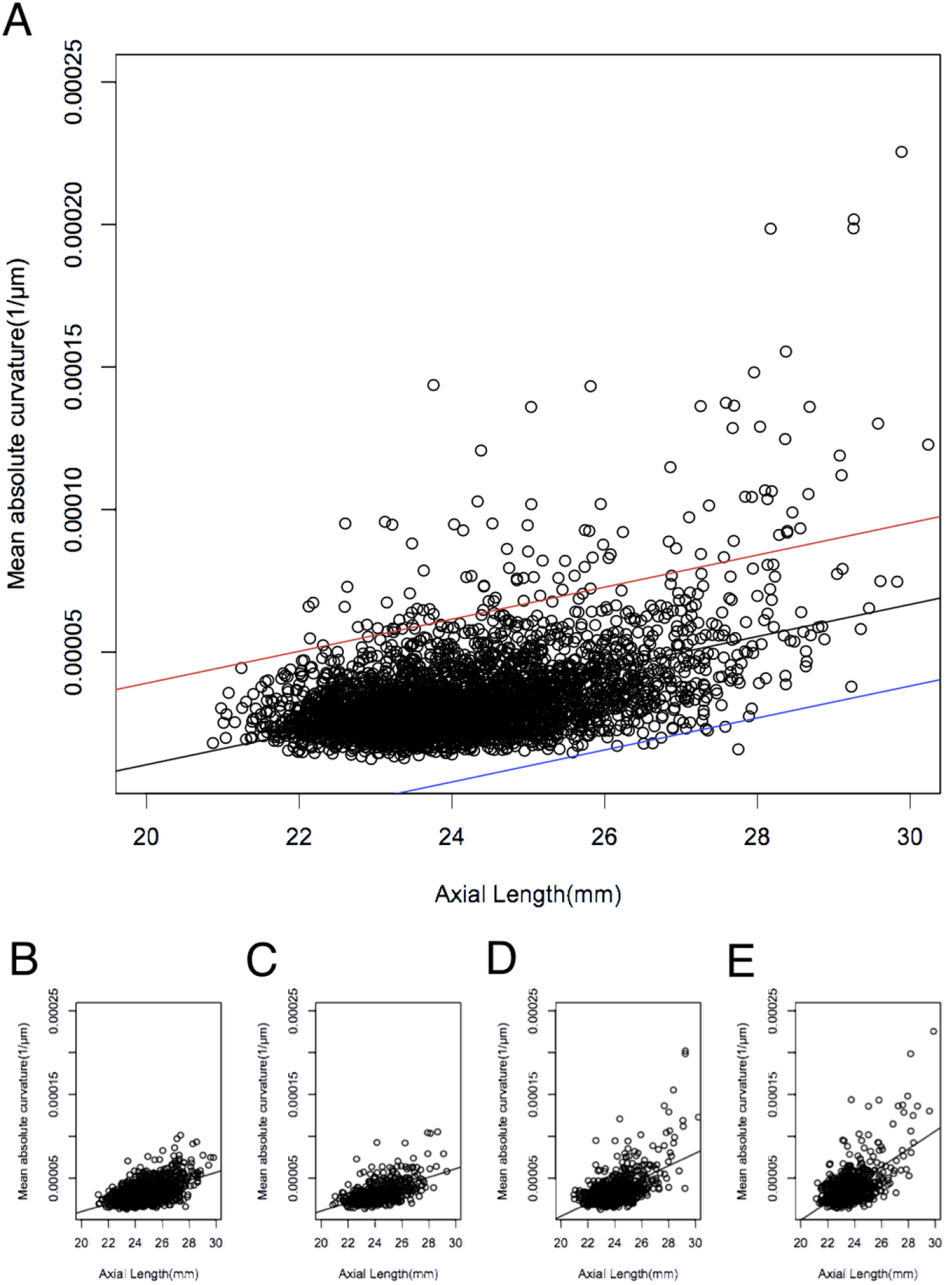
Scatter plot of studied eyes assigning axial length to the horizontal axis and mean absolute curvature to the vertical axis. (**A**) All 3748 eyes are plotted with linear regression line (black) and 95% confidence intervals of lower (blue) and upper (red) lines. The scatter plots were stratified by age (B, < 50 years; C, 50–59 years; bottom D, 60–69 years; E, ≥ 70 years).

A significant correlation was found between mean curvature and AL (*P* = 2 × 10^−16^, *R* = 0.480; Fig. 4). To evaluate the effect of age on this relationship, the scatter plot was stratified by age group. Mean curvature and AL showed a significant correlation in all age groups (*P* < 2.2 × 10^−16^). For every 1 mm elongation of AL, mean curvature increased by 4.7 × 10^−6^, 5.06 × 10^−6^, 7.54 × 10^−6^, and 10.6 × 10^−6^ in subjects aged 35–49 years, 50–59 years, 60–69 years, and 70–79 years, respectively. These correlations were significantly affected by age (*P*[interaction] < 2 × 10^−16^).

## Discussion

In the present study, we evaluated the prevalence of posterior staphyloma in the Japanese population using fundus photography and OCT in 3748 participants in the community-based cohort of the Nagahama Study. The prevalence of posterior staphyloma was 2.05% in this cohort, and increased with age from 0.53% in subjects aged 35–49 years to 4.02% in those aged 70–79 years. Although the male study participants were slightly older (59.4 ± 13.8 years vs 56.4 ± 13.4 years) and had slightly longer AL (24.43 ± 1.35 mm vs 24.00 ± 1.43 mm), there was no significant difference in the prevalence of staphyloma between men and women. The overall prevalence of staphyloma in highly myopic eyes was 10.9%, but increased with age from 2.7% in subjects aged 35–49 years to 43.6% in those aged 70–79 years. Thus far, the prevalence of staphyloma has not been thoroughly investigated in association with age or AL using OCT in cohort studies. Quantitative analysis of posterior fundus shapes of eyes with staphyloma measuring local curvature on OCT images was quite useful for demonstrating the characteristic posterior fundus shapes of eyes with staphyloma according to sex, age, and AL. Both mean curvature and variance of curvature in eyes with staphyloma were greater in female, elderly, and highly myopic participants in our study, indicating that posterior fundus shapes are more severe and less smooth in these groups. Further, quantitative evaluation of OCT images would enable eyes with staphyloma to be detected in a large-scale cohort study using cutoff values for mean curvature and variance of curvature. Further studies in other cohorts are warranted for refinement and standardization of the methodologies.

The prevalence of high myopia in the present cohort of the Nagahama Study is comparable with that in a previously reported Japanese population-based cohort from the Tajimi Study^4^ **(**Table S3**)**. Considering that the Tajimi Study was conducted between 2000 and 2001 and our present study was performed between 2013 and 2015, the prevalence of high myopia should be similar between the group aged 40–49 years in the Tajimi Study and the group aged 50–59 years in our present study, the group aged 50–59 years in the Tajimi Study and the group aged 60–69 years in our study, and the group aged 60–69 years in the Tajimi Study and the group aged 70–79 years in our study. The similar prevalence of high myopia between these two studies after adjusting for the nearly one decade time gap suggests that the distribution of refractive error/AL is not seriously biased in the present study, in spite of its community-based cohort nature.

The prevalence of posterior staphyloma in our cohort is comparable with but slightly higher than that previously reported in population-based studies. Our finding of no difference in the prevalence of staphyloma between men and women is in agreement with previous reports. The prevalence of posterior staphyloma in our study (2.05%) is slightly higher than the reports of 1.6% in the Beijing Eye Study^23^, 0.8% in the Handan Eye Study^22^, and 0.7% in the Blue Mountains Eye Study^21^. The difference could be explained by differences in the methodologies used to detect posterior staphyloma and by the prevalence of high myopia in the study cohorts. Previous studies relied on subjective clinical examination using ophthalmoscopy or two-dimensional fundus photographs, such that backward bowing of the posterior pole could not be assessed adequately in eyes with slight staphyloma, and this methodological limitation has been frequently argued. In the present study, we examined both fundus photographs and OCT images, so we may have detected staphyloma that might be missed on ophthalmoscopy and/or fundus photographs alone. With regard to the prevalence of high myopia, the rate of eyes with ≤ −5 D refractive error was 3.3% in the Beijing Eye Study^23^, 2.1% in the Handan Eye Study^22^, and 2.7% in the Blue Mountains Eye Study^21^, but is much higher at 13.3% in our present study. We speculate that the much higher prevalence of high myopia in our study is attributed to the differences in mean age of the participants and in the periods when the studies were performed. The mean age of the cohort of the present study is slightly younger than that of the previous large cohort studies (57.3 ± 13.6, 60.4 ± 10.0, and 66.2 years in the present study, the Beijing Eye Study^23^, and the Blue Mountains Eye Study, respectively). The Beijing Eye Study and Blue Mountains Eye Study were conducted in around 2000 (in 2006 and 1994–1997, respectively) and our present study was performed between 2013 and 2015. Considering that the prevalence of high myopia has increased markedly worldwide in younger generations, it is natural that our younger cohort includes more highly myopic participants than the previous cohort studies. Consequently, given that the prevalence of staphyloma increases as myopia becomes more severe, the higher prevalence rate of staphyloma in the present study would result from the higher prevalence rate of high myopia.

The prevalence of posterior staphyloma was 10.9% among the highly myopic eyes in our study, which seems lower when compared with the calculated prevalence rates for posterior staphyloma in highly myopic eyes that vary from 19% to 90% in previous studies **(**Table S1**)**. Most of the previous studies of posterior staphyloma in highly myopic eyes in Table S1 were hospital-based (mainly high myopia clinic-based) and population-based research is very limited. It is thought that many patients with staphyloma do not visit hospitals until they are aware of a visual disturbance, which results in longer AL and a higher prevalence rate of posterior staphyloma among highly myopic eyes in hospital-based studies.

Quantitative evaluation of OCT images would be useful in a cohort study to evaluate the prevalence of eyes with posterior staphyloma and investigate its association with sex, age, and AL. In the present study, detection of eyes with posterior staphyloma using measured values for mean curvature (≥6.97 × 10^−5^) and variance of curvature (≥2.06 × 10^−9^) showed fairly good sensitivity (84.4%) and specificity (99.7%). Detection of eyes with staphyloma using quantitative OCT measurement might be able to allow comparison of the prevalence of staphyloma and its correlations with sex, age, and AL between the large cohort studies.

Graphs plotting the mean curvature and variance in curvature of each eye could show the morphologic characteristics of posterior fundus shapes of eyes with posterior staphyloma. Our present study clearly demonstrated that the posterior fundus curvature of eyes with staphyloma became more severe and less smooth in female, elderly, and highly myopic participants. In spite of the similar prevalence of staphyloma between men and women, the tendency for women to develop more severe and less smooth staphyloma means that women with high myopia are likely to visit the hospital more frequently than men, which would be the reason why most hospital-based studies have included more female patients when investigating the complications of high myopia.

Quantitative evaluation of posterior fundus shapes of eyes with staphyloma enabled us to analyze the effect of age and AL on the development of staphyloma. The severity of posterior part of staphyloma, represented by mean curvature, was significantly correlated with AL. For every 1 mm elongation of AL, mean curvature increased by 4.7 × 10^−6^, 5.06 × 10^−6^, 7.54 × 10^−6^, and 10.6 × 10^−6^ in subjects aged 35–49, 50–59, 60–69, and 70–79 years, respectively. Hsiang *et al*. also showed a higher prevalence of posterior staphyloma in subjects with high myopia who were aged 50 years or older than in their counterparts who were younger than 50 years, although the AL was not significantly different between these two age groups^16^. The likelihood of a longer AL facilitating the development of staphyloma would increase with age.

It is well known that staphyloma facilitates progression of myopic complications, and eyes with staphyloma have significantly worse visual acuity if high myopia is present^29,30^. Quantitative analysis of fundus shape would lead not only to a better understanding of the pathogenesis of staphyloma but also to prediction of its development and progression, as well as give an indication of the prognosis for vision-threatening myopic retinopathies in each patient with high myopia at risk. Previous studies have attempted quantitative analysis of the association between staphyloma and myopic retinopathy by measuring the depth of staphyloma using B-scan ultrasonography^31^, measuring staphyloma depth using OCT^17^, measuring best-fit radius of curvature^32^, or classifying posterior staphyloma^18^. However, grading of B-scan ultrasonography is not suitable for detailed analysis of staphyloma. The measured depth of staphyloma substantially varies depending on the scope of OCT examination. The best-fit radius for an OCT image cannot represent a detailed shape of the fundus curvature because the shape of staphyloma is not a perfect circle. Our quantitative measurement of local curvature on OCT images can overcome such weakness of the previously reported methods and evaluate the shape of the posterior pole in detail. Considering the increase in prevalence of myopia and high myopia in the younger age groups, the prevalence of myopic retinopathies such as posterior staphyloma, myopic traction maculopathy, choroidal neovascularization, and diffuse/patchy chorioretinal atrophy will increase dramatically in the upcoming decades. Further quantitative analysis of local curvature on OCT should be performed to prevent the development of these vision disorders.

This study has certain limitations. First, because of its community-based cohort nature, it could not provide a population-based prevalence of posterior staphyloma. However, as mentioned above, the distribution of AL/refractive error seemed to be compatible with that in a previously reported large cohort study in Japan. Furthermore, owing to its relatively large cohort size, we could determine the association between the characteristics of staphyloma and age, sex, and AL. Second, this study was cross-sectional in design, which precluded analysis of causation. However, given that the ten-year follow-up of the Nagahama Study is scheduled for 2018–2022, further longitudinal investigation will enable us to analyze changes in AL, mean curvature, variance in curvature, and their associations over time in more detail. Third, the 6 mm OCT scan width applied in this study is not wide enough to include the entire edge of the staphyloma, especially in eyes with widely extended staphyloma. Our method might lead to underestimation of posterior staphyloma. According to the widely accepted definition of staphyloma as an outpouching of the wall of the eye that has a radius of curvature less than the surrounding curvature of the wall of the eye^26^, the curvature within the staphyloma must be steeper than that of the surrounding curvature. To detect this alteration in curvature, detecting the outer margin of staphyloma using ultra-wide field imaging of the fundus, such as the Optos scanning laser ophthalmoscope, is a reasonable method, but ultra-wide field imaging has not been performed in most cohort studies. Using OCT is another option to detect the alteration in curvature. OCT can easily analyze the curvature at the macular area and our previous study clearly demonstrated that analysis of curvature with OCT could differentiate eyes with staphyloma from eyes without staphyloma^19^. In the present study, we evaluated staphyloma using fundus photography according to previous cohort studies with OCT images used in a supplemental manner, and the prevalence of eyes with staphyloma was comparable with that in previous reports. The recent cohort studies have often used OCT but not ultra-wide field imaging of the fundus as yet. Considering the availability of OCT, evaluation of posterior fundus shapes of eyes with staphyloma using fundus photography and 6 mm OCT should be a reasonable approach in cohort studies.

In conclusion, our study of 3748 subjects aged 35 years or older has provided the first evidence concerning the prevalence of posterior staphyloma and quantitative characteristics of posterior fundus shapes of eyes with posterior staphyloma by using fundus photographs and OCT images to analyze fundus shapes stratified by age, sex, and AL in the Japanese population. Quantitative analysis of posterior fundus shapes of eyes with staphyloma measuring local curvature on OCT images was quite useful and showed clearly that both mean curvature and variance of curvature in eyes with staphyloma were greater in female, elderly, and highly myopic participants. The values of ≥6.97 × 10^−5^ for mean absolute curvature and ≥2.06 × 10^−9^ for variance of absolute curvature had fairly good sensitivity and specificity for detection of eyes with staphyloma and would help us to compare the prevalence of staphyloma between the large cohort studies with more refinement and standardization of methodologies in the future. Considering the markedly increased prevalence of high myopia in the younger age groups, the prevalence of myopic retinopathies will inevitably increase in the upcoming decades. Further data-accumulation and quantitative analysis of posterior eye shape by measurement of local curvature on OCT images in other cohort studies would make it possible to discuss the numerical definition of posterior staphyloma, and that would lead to the further detailed analyses such as correlations between the severity of the fundus shapes and the vision-threatening myopic complications in efforts to prevent these common vision disorders.

## Electronic supplementary material

Dataset 1
