# Supplementary material for: Prevalence of posterior staphyloma and factors associated with its shape in the Japanese population
Source: Sci Rep. 2018 Mar 15;8:4594. doi: 10.1038/s41598-018-22759-y (PMC5854606; doi:10.1038/s41598-018-22759-y)
Supplement: Supplementary file 1 — Dataset 1 [file 41598_2018_22759_MOESM1_ESM.docx]

**Prevalence of posterior staphyloma and factors associated with its shape in the Japanese population**

Shogo Numa, Kenji Yamashiro, Tomotaka Wakazono, Munemitsu Yoshikawa, Masahiro Miyake, Hideo Nakanishi, Akio Oishi, the Nagahama Study Group, Yasuharu Tabara, Fumihiko Matsuda, Nagahisa Yoshimura, Akitaka Tsujikawa

**Figure S1.** (A) Receiver-operating characteristic (ROC) curve for mean absolute curvature and (B) ROC curve for variance of absolute curvature to detect eyes with posterior staphyloma.


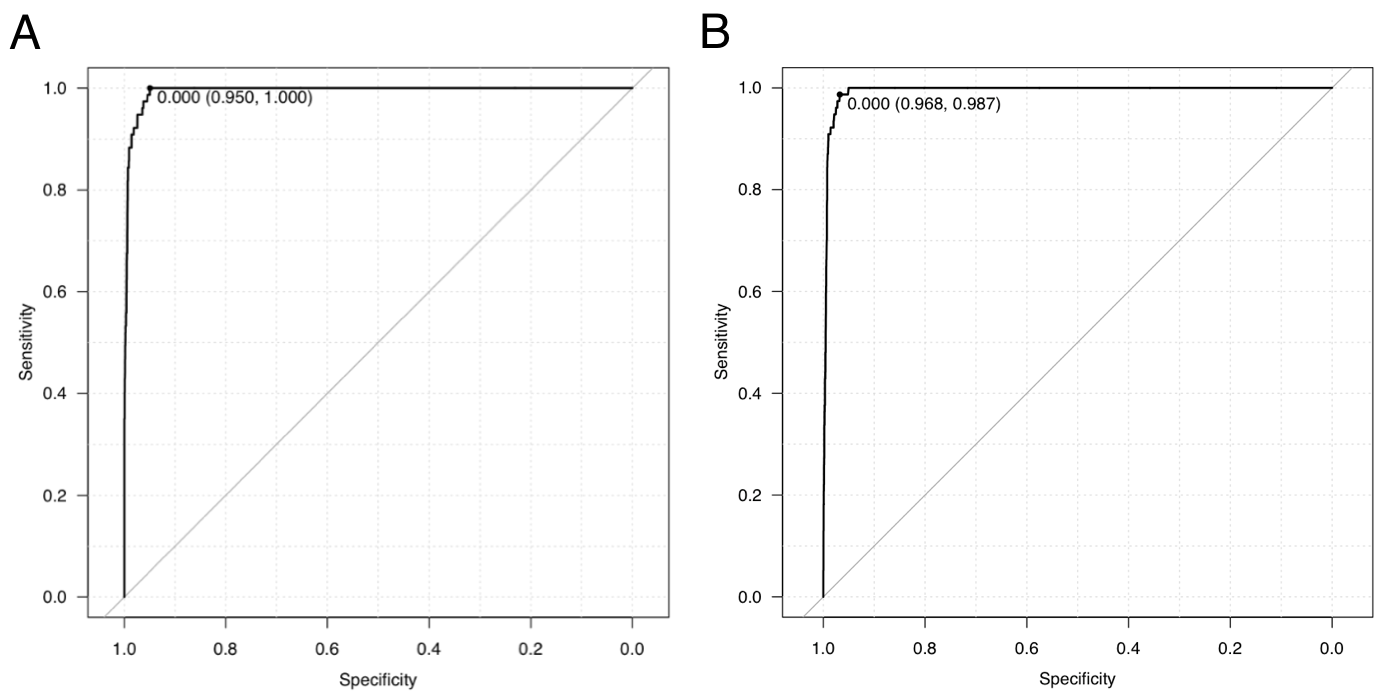


**Table S1.** Prevalence of posterior staphyloma among highly myopic subjects in the previous studies

| Reference | Prevalence | n | Definition | Age, years | AL | -based |
| --- | --- | --- | --- | --- | --- | --- |
| Curtin et al^1^ | 19% | 538 | ≥26.5 mm | - | - | Myopia clinic- |
| Chang et al^2^ | 23% | 332 | ≤ -6 D | 51.5 | - | Population- |
| Koh et al^3^ | 32% | 593 | <-6 D | 21.1±1.2 | 27.45±1.2 | Population-  stratified sampling |
| Henaine-Berra et al^4^ | 35% | 116 | < -8 D | 36.9±15.9 | 28.9±2.3 | Hospital |
| Grossniklaus et al^5^ | 35% | 308 | - | 59 | 26.9 | Autopsy |
| Shen et al^6^ | 39% | 33 | ≤ -6 D and ≥ 25 mm | 27.4±12.8 | - | Hospital |
| Ohno-Matsui et al^7^ | 51% | 198 | ≤ -8 D or ≥ 26.5 mm | 64.3±11.5 | 30.0±2.3 | HM clinic |
| Hsiang et al8 | 58% | 134 | ≤ -8 D | 54.1 | 29.13 | HM clinic |
| Moriyama et al^9^ | 65% | 321 | ≤ -6 D or ≥26.5 mm | 47.2±15.6 | 29.2±1.8 | HM clinic |
| Hayashi et al^10^ | 66% | 806 | ≤ -8 D or ≥26.5 mm | 41.1±16.7 | 28.7±1.9 | HM clinic |
| Kaneko et al^11^ | 67% | 115 | ≤ -8 D or ≥26.5 mm | 60.1±13.0 | 29.8±2.2 | HM clinic |
| Steidl et al^12^ | 76% | 116 | ≤ -3 D | 42.5±16.9 | 29.8 | Myopia clinic |
| Ohno-Matsui et al^13^ | 84% | 488 | ≤ -8D or ≥26.5 mm | 57.1±13.8 | 29.9±2.0 | HM clinic |
| Baba et al^14^ | 90% | 209 | ≤ -8D or ≥26.5 mm | 55.0±14.9 | 29.3±1.9 | HM clinic |
| Present study | 11% | 395 | >26 mm | 50.4±12.4 | 27.0±0.9 | Population |

n, number of studied eyes; AL, axial length; HM, high myopia

**Table S2.** Characteristics of all study participants.

|  | Total | Men | Women |
| --- | --- | --- | --- |
| n | 3748 | 1193 | 2555 |
| Age (years) | 57.3±13.6 | 59.4±13.8 | 56.4±13.4 |
| ≤49 | 1317 (35.1%) | 367 (30.8%) | 950 (37.2%) |
| 50–59 | 529 (14.1%) | 135 (11.3%) | 394 (15.4%) |
| 60–69 | 1006 (26.8%) | 317 (26.6%) | 689 (27.0%) |
| ≥70 | 896 (23.9%) | 374 (31.3%) | 522 (20.4%) |
| AL (mm) | 24.14±1.42 | 24.43±1.35 | 24.00±1.43 |
| <26 | 3353 (89.5%) | 1034 (86.7%) | 2319 (90.8%) |
| 26–28 | 340 (9.1%) | 137 (11.5%) | 203 (7.9%) |
| 28–30 | 53 (1.4%) | 22 (1.8%) | 31 (1.2%) |
| ≥30 | 2 (0.05%) | 0 | 2 (0.1%) |

AL, axial length

**Table S3.** Prevalence of high myopia in a previous population-based cohort in Japan (Tajimi Study) and in the present study (Nagahama Study).

| Age  group  (years) | Tajimi Study in 2000–2001 | | | Nagahama Study in 2013–2015 | | |
| --- | --- | --- | --- | --- | --- | --- |
|  | High myopia (< -5.0 D) | | | High myopia (< -5.0 D) | | |
|  | Whole | Male | Female | Whole | Male | Female |
| ≤49 | - | 17.7% | 15.0% | 20.5% | 20.1% | 20.6% |
| 50–59 | - | 8.7% | 7.1% | 17.1% | 20% | 16.1% |
| 60–69 | - | 3.0% | 4.4% | 7.7% | 5.2% | 8.8% |
| 70–79 | - | 0% | 3.0% | 3.3% | 2.9% | 2.6% |
| 80+ | - | 0% | 4.3% | - | - | - |
| Total | 8.2% | 8.3% | 8.1% | 13.3% | 10.7% | 13.0% |

**References**

1. Curtin BJ, Karlin DB. Axial length measurements and fundus changes of the myopic eye. Ⅰ. The posterior fundus. *Tran.s Am. Ophthalmo.l Soc.* **68**, 312-34 (1970)
2. Chang L, et al. Myopic-related fundus changes in Singapore adults with myopia*. Am. J. Ophthalmol.* **155**, 991-999 (2013)
3. Koh V, et al. Myopic maculopathy and optic disc changes in highly myopic young Asian eyes and impact on visual acuity. *Am. J. Ophthalmol.* **164**, 69-79 (2016)
4. Henaine-Berra A, et al. Prevalence of macular anatomic abnormalities in high myopia. *Ophthalmic Surg. Lasers Imaging Retina* **44**, 140-144 (2013)
5. Grossniklaus HE, Green W. Pathologic findings in pathologic myopia. *Retina* **12**, 127-133 (1992)
6. Shen P, Zheng Y, Ding X, et al. Biometric measurements in highly myopic eyes. *J. Cataract Refract. Surg.* **39**, 180-187 (2013)
7. Ohno-Matsui K. Proposed classification of posterior staphyloma based on analysis of eye shape by three-dimensional magnetic resonance imaging and wide-filed fundus imaging. *Ophthalmology* **121**, 1798-1809 (2014)
8. Hsiang HW, et al. Clinical characteristics of posterior staphyloma in eyes with pathologic myopia. *Am. J. Ophthalmol.* **146**, 102-110 (2008)
9. Moriyama M, et al. Morphology and long-term changes of choroidal vascular structure in highly myopic eyes with and without posterior staphyloma. *Ophthalmology* **114**, 1755-1762 (2007)
10. Hayashi K, et al. Long-term pattern of progression of myopic maculopathy: a natural history study. *Ophthalmology* **117**, 1595-1611 (2010)
11. Kaneko Y, et al. Areas of nonperfusion in peripheral retina of eyes with pathologic myopia detected by ultra-widefield fluorescein angiography. *Invest. Ophthalmol. Vis. Sci*. **55**, 1432-1439 (2014)
12. Steidl SM, Pruett RC. Macular complications associated with posterior staphyloma. *Am. J. Ophthalmol.* **123**, 181-187 (1997)
13. Ohno-Matsui K, et al. Association between shape of sclera and myopic retinochoroidal lesions in patients with pathologic myopia. *Invest Ophthalmol Vis Sci* **53**, 6046-6061 (2012)
14. Baba T, et al. Prevalence and characteristics of foveal retinal detachment without macular hole in high myopia. *Am J Ophthalmol* **135**, 338-342 (2003)
